# Recognising ethnocultural diversity in chronic pain assessment: validation of the Pictorial Representation of Illness and Self Measure (PRISM) for use with culturally diverse communities

**DOI:** 10.1186/s12955-019-1126-9

**Published:** 2019-04-08

**Authors:** Bernadette Brady, Irena Veljanova, Toni Andary, Troy Southwell, Lucinda Chipchase

**Affiliations:** 1 School of Science and Health, Western Sydney University, Sydney, NSW Australia; 2 Departments of Pain Medicine and Physiotherapy, Liverpool Hospital, Locked Bag 7103, Liverpool, NSW 1871 Australia; 3 School of Social Science and Psychology, Western Sydney University, Sydney, NSW Australia; 4 Physiotherapy Department, Fairfield Hospital, Fairfield, NSW Australia

**Keywords:** Chronic pain, Cultural diversity, Pain measurement, Suffering

## Abstract

**Background:**

A comprehensive and accurate assessment of pain is critical for successful pain management. However, there is a lack of reliable and valid assessment tools for exploring multidimensional aspects of the chronic pain experience in culturally and linguistically diverse communities. This study investigates the reliability and validity of the Pictorial Representation of Illness and Self Measure + (PRISM+) for evaluating pain-related suffering and the sociocultural context of chronic pain within culturally and linguistically diverse patient cohorts.

**Method:**

Three prospective validation studies are reported for three culturally and linguistically diverse communities. Two hundred and fifty-one patients with chronic pain who self-identified as Assyrian (*n* = 85), Arabic (*n* = 83) or Vietnamese (*n* = 83) completed a PRISM+ assessment, alongside a battery of standardised pain assessments. To evaluate construct validity, the position of the ‘pain’ disk placement was correlated with the Brief Pain Inventory (BPI), Depression Anxiety and Stress Scale (DASS), and the Short-Form 36 Health Survey (SF-36). For content validity, thematic analysis of patient narratives accompanying each disk placement was conducted. Test-retest reliability of repeated ‘pain’ and five additional disks (PRISM+) values was analysed using intra-class correlation coefficients.

**Results:**

The PRISM pain assessment demonstrated moderate to good test-retest reliability for Arabic (ICC 0.76; 95% CI 0.65–0.84), Assyrian (ICC 0.65; 95% CI 0.50–0.76) and Vietnamese (ICC 0.82; 95% CI 0.73–0.88) patients. Moderate correlations between the PRISM ‘pain’ disk and sub-scores for the BPI, DASS and SF-36 were found (*p* < 0.001). Patient interpretations of the ‘pain’ disk aligned with accepted definitions of suffering, supporting content validity for PRISM. For the additional disks (PRISM+), moderate to good test-retest reliability (ICC 0.67–0.88) was observed and qualitative analysis highlighted each disk reflected social and cultural values.

**Conclusion:**

The PRISM demonstrates acceptable psychometric properties for measuring pain-related suffering for participants with chronic pain across three culturally and linguistically diverse communities. The use of additional disks (PRISM+) presents a reliable and valid option for exploring social and cultural dimensions of chronic pain in clinical encounters.

**Electronic supplementary material:**

The online version of this article (10.1186/s12955-019-1126-9) contains supplementary material, which is available to authorized users.

## Introduction

Contemporary chronic pain assessments seek to holistically appraise biomedical, psychosocial and behavioural contributors to the pain and disability experience, often with patient-reported measures [1]. While a multitude of reliable and valid questionnaires explore biopsychosocial dimensions of chronic pain, their application in culturally and linguistically diverse (CALD) communities is thwarted by challenges achieving cross-cultural equivalence and robust psychometric properties [2, 3]. Further, a critical element missing within standardised pain assessments is the personal, social and cultural narrative of pain [4, 5]. Eliciting such narratives during cross-cultural encounters between healthcare providers and patients is fundamental for arriving at shared understandings of pain, and for guiding management [6, 7]. As such, there is a need for tools that combine patient narratives with structured measurement of physical, psychological and sociocultural dimensions of pain, for CALD communities.

One measurement tool, combing patient narrative and structured measurement of multiple illness dimensions is the Pictorial Representation of Illness and Self Measure (PRISM) [8, 9]. The PRISM quantitatively measures the subjective position of the patient’s illness in relation to self, while the therapist simultaneously elicits a narrative of how illness influences a patient’s identity [8, 9]. This is achieved by the patient placing a coloured disk representing ‘illness’ on a ‘life’ board, in a position that symbolises the effect of illness on the integrity of the person, and their sense of self. Patients can be encouraged to describe their reasoning while they place the disk, which generates a succinct illness narrative [10, 11].

In an extension of the original PRISM, PRISM+ utilises multiple coloured disks to represent other important aspects of the patient’s life (e.g. family, work). Each disk can be applied to the ‘life’ board to reflect relationships between illness and other aspects of the patient’s life [9, 12]. Thus, PRISM+ can explore illness in a biopsychosocial context by traversing multiple dimensions, and as such, it has promise for chronic pain research and clinical practice.

Since inception as a measure of coping with chronic illness, PRISM has been validated as a tool for measuring illness-related suffering [8, 13–17]. In chronic disease settings, the primary quantitative score of PRISM, and the illness narratives it elicits, scope common definitions of suffering [8, 18, 19]. Cassell (1999) defines suffering as a state of distress arising from a threat or disruption to the integrity of a person and their sense of self [20]. Qualitative evidence arising from PRISM studies highlights that patients consistently appraise the ‘illness’ disk placement according to perceived threat to personhood/self [11, 21, 22]. Similarly, among the multiple conceptualisations of suffering in the literature are four common themes that can all be explored within the PRISM [8, 23]. Specifically, suffering i) is holistic and multi-dimensional, ii) is associated with physical symptoms (e.g. pain), iii) includes psychological distress (e.g. depression, anxiety) and iv) includes existential dimensions (e.g. meaning of life) [23]. Consistent with this broad definition, significant correlations have been consistently observed in chronic disease cohorts between the quantitative ‘illness’ score and physical and psychological symptoms [14, 15, 17, 21, 22, 24–28]. Collectively, these findings provide support for the use of PRISM as a measure of illness related suffering.

As a measure of illness related suffering, PRISM+ has potential clinical utility in chronic pain clinical practice and research [12]. In a cohort of 22 participants, Kassardjian et al. [12] demonstrated high test-retest reliability (r = 0.98), and, among 124–130 participants, weak-moderate correlations with other commonly used pain assessment tools (pain intensity, quality of life and pain catastrophising scales). Further, content validity, derived from 26 participant responses, associated PRISM ‘pain’ and four additional disks with biopsychosocial aspects of pain [12]. These promising findings warrant further investigation, particularly in CALD cohorts. Specifically, PRISM’s transcendence of numeric and constrained verbal descriptors, accounts for differences in communication, pain expression and literacy [6, 29] that have been barriers to personal, social and cultural narratives of pain in CALD groups. As such, PRISM may be an accessible alternative measure of biopsychosocial pain dimensions in CALD communities.

The aim of this study was to investigate reliability and validity (construct and content) of the PRISM+ for evaluating i) pain-related suffering and ii) the sociocultural context of chronic pain; in three CALD communities living in Australia: Assyrian, mixed Arabic and Vietnamese communities. Consistent with other uses of the PRISM+ [12, 14, 15], it was hypothesised that the ‘pain’ disk would have adequate test-retest reliability across three CALD communities, and it would correlate with other dimensions of chronic pain (pain intensity, pain-related disability, quality of life and emotional functioning). For the ‘additional disks’, no specific hypothesis was generated and the approach was explorative for reliability and content validity.

## Methods

### Participants

This multicentre validation study was conducted across four public hospitals in South West Sydney, Australia (Liverpool, Fairfield, Bankstown and Auburn). Consecutive consenting adults (> 18 years of age) from those referred for physiotherapy or pain clinic treatment for a neuromusculoskeletal chronic pain condition (confirmed on clinical assessment and of greater than 3 months duration) were invited to participate if they identified as a first-generation member of Assyrian, Arabic speaking or Vietnamese communities. There were no specific exclusion criteria.

### Pictorial representation of illness and self measure + (PRISM +)

This study utilised a paper version of the PRISM+ [11], laminated to allow disks to be moved and positioned easily by patients. The original paper version was selected over electronic versions as our experience with CALD communities in South West Sydney Local Health District (SWSLHD) indicated high rates of social disadvantage and limited familiarity with technology in health [30]. Further, the paper versions allowed for translations to be readily printed and taken home by participants for repeat testing.

The PRISM comprises a white A4 page (210 × 297 mm) with a fixed 7-cm yellow diameter circle printed in the bottom corner that represents the participant’s ‘self’ (Additional file 1). The participant is asked to “imagine that this white template represents your life as it is now” and that "the yellow disk in the bottom corner represents your ‘self’ [21]. Participants are handed a smaller (5-cm diameter) red disk representing their ‘pain’ and they are asked to place the disk on the page in a position that best reflects the position of pain in their life [21]. Additional prompts regarding ‘the importance’ or ‘intrusiveness’ of pain in their life, relative to the ‘self’ were offered as needed, consistent with previous applications of the PRISM+ [12]. The distance between the centre of the yellow ‘self’ circle and the centre of the red ‘pain’ circle to the nearest mm, termed the ‘self-pain separation’ (SPS) was measured (range 0-27 cm), with lower SPS scores (distances in cm) reflecting higher perceived suffering due to pain [12, 14].

For the PRISM+, the additional disks selected were informed by research involving Assyrian, Arabic and Vietnamese communities from SWSLHD [6, 30], and previous use of the PRISM+ in chronic pain settings [12]. Previous qualitative research findings from each CALD community emphasised the importance of family, fulfilling traditional occupational roles, social relationships, and spirituality, on the experience of pain and construction of ethnocultural identity [6]. As such, these dimensions were included as part of a holistic pain assessment. Therefore, five supplemental disks (5-cm diameter) that represented other aspects of a participant’s life including ‘spouse/partner’ (purple), ‘family’ (green), ‘recreation’ (black), ‘work’ (blue), and ‘spirituality’ (grey), were incorporated. The participant was asked to place each disk on the page, in a position that corresponded to the position of that additional disk in their life (if applicable to their life), and its importance, relative to the ‘self’. A ‘self-disk-separation’ measure was scored for all five additional disks, calculated as the distance between the centre of the yellow ‘self’ circle and the centre of the corresponding disk circle (0-27 cm).

### Translation and adaptation of the PRISM+

The pictorial nature of the PRISM+ reduces the potential for mistranslation of the instrument [15]. Despite this, translation and cross-cultural adaptation processes were undertaken [31]. Forward translation of the PRISM+ instructions and words into Assyrian, Arabic and Vietnamese was completed by an accredited National Australian Authority for Translators and Interpreters (NAATI) translator. A second independent translation was also conducted by three bilingual health professionals or interpreters, experienced in chronic pain management. The two translations for each language were compared and synthesised into one document [31]. Backwards translation was then completed by another two NAATI accredited translators and interpreters. Reconciliation was achieved by comparing the source PRISM+ to the translated PRISM+ [31, 32].

The resulting tool was piloted with ten participants with chronic pain from each target CALD community. Two important considerations arose during pilot testing. First, participants and administering therapists recommended administering the additional disks prior to the ‘pain’ disk to facilitate conceptual interpretation of the task. Second, none of the Assyrian participants could read the Assyrian language, preventing them from using the Assyrian PRISM+ or translated research documents (i.e. participant information sheets). Rather, and according to their preference, all Assyrian participants read the Arabic translation, which reflected the historically limited opportunities for education in their ethnocultural language in their home country [33] (*p 105*). Nevertheless, all Assyrian participants expressed a preference for verbal communication and explanation of the task in Assyrian. As such, a decision was made to continue with a validation study, using Assyrian verbal explanations alongside participant choice of the written language tool (colour coded Arabic or English versions as desired).

### Procedure

**Fig. 1:**
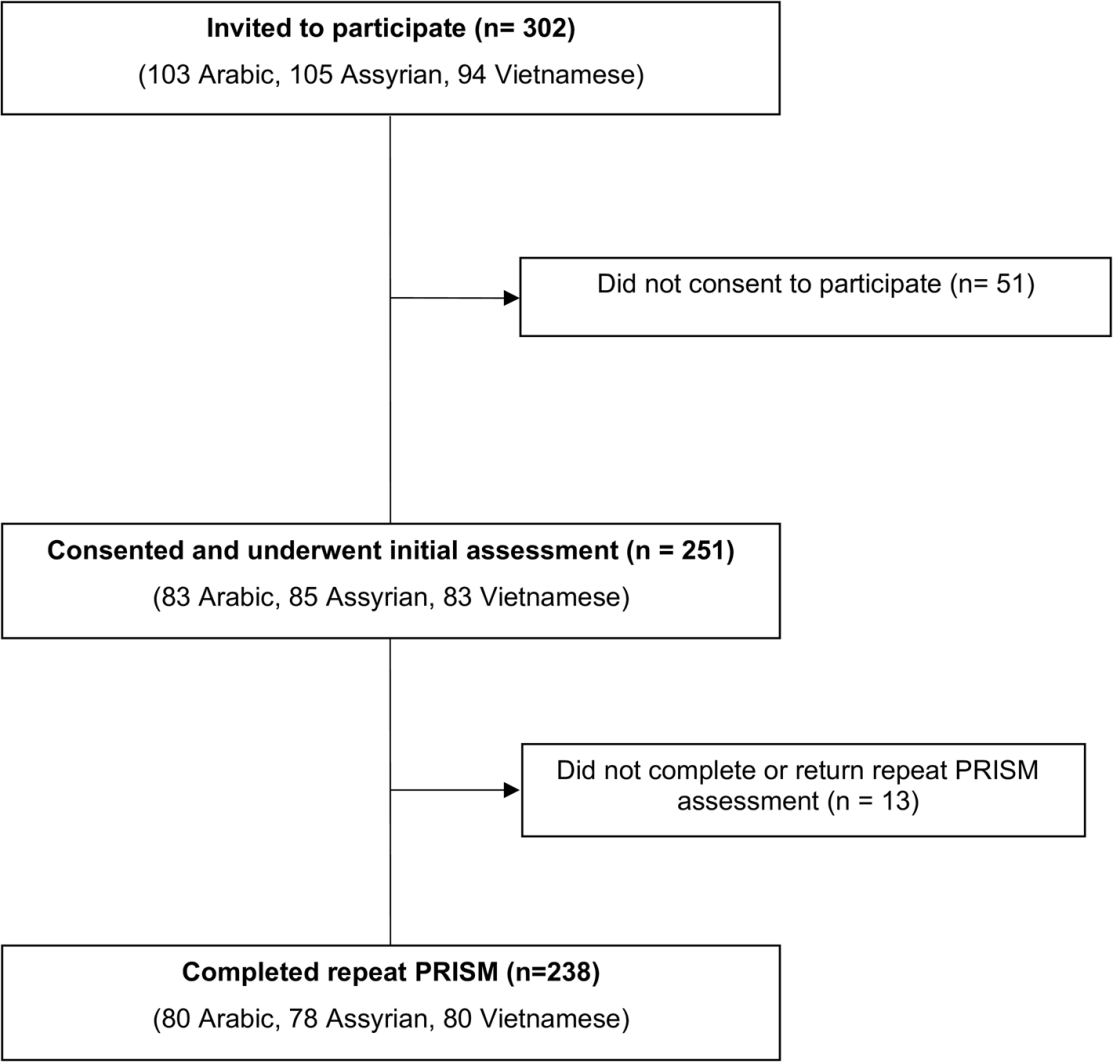
Flow of Participant Recruitment

Between November 2015 and April 2017, 302 participants were invited to participate (Fig. 1). In each participating hospital, a senior physiotherapist identified potential participants from physiotherapy or pain clinic waitlists. Participants were given information about the study prior to, or after, their clinic appointment. All consenting participants completed a single face to face assessment with a physiotherapist that included a short demographic interview. Each participant then completed the PRISM+, the Brief Pain Inventory (BPI), the Short-Form-36 (SF-36), and the short form Depression, Anxiety and Stress Scale (DASS-21) in their preferred written language. For Assyrian participants, Arabic or English versions of standardised questionnaires were used because Assyrian translations were not available, nor preferred by participants. While not specifically measuring pain-related suffering, these tools explored an aspect of the conceptualisation of suffering as theorised by Schultz et al. [23] and had been psychometrically tested in Arabic and Vietnamese populations. For the PRISM+ task, participant responses for each disk placement were documented verbatim at the time of the interview for the first 50 participants from each community. All participants consented to complete the PRISM+ assessment on a second occasion 24–48 h later, via face to face interview or phone interview with a second investigator. For phone interview, participants were given a PRISM+ tool to take home and a telephone appointment was made at a time of day that best matched the time of the initial assessment. Participants were then telephoned by the second investigator and guided through disk placement, with disks secured to the laminated tool by adhesive gum. Participants placed completed tools in a sealed A4 envelope that they returned either via post or at their next scheduled appointment (if within a week). Scoring was completed by the investigator when the envelope was returned. All participants were asked to reflect if their pain had substantially changed from their initial assessment prior to completing the second assessment.

### Brief pain inventory (BPI)

The BPI is a short, self-administered 11-item numerical rating questionnaire that measures the intensity (Pain Severity Scale), and the interference (Pain Interference Scale) caused by pain [34]. The BPI has demonstrated reliability and validity for patients with chronic pain [35, 36] and Arabic and Vietnamese translations have undergone psychometric testing in settings/conditions other than chronic neuromusculoskeletal pain [35, 37]. Scores from the four pain items (worst, least, average, and right now) are averaged to ascertain Pain Severity scores, while Pain Interference is calculated from the seven functional activity measures (general activity, mood, walking ability, normal work, relationships with other people, sleep, and enjoyment of life). All items (including the two subscales) are measured on a scale from 0 (representing “no pain” or “no interference”) to 10 (representing “pain as bad as you can imagine” or “complete interference”) [34].

### The SF-36

The SF-36 questionnaire is a widely used, validated measure of health-related quality of life, consisting of 36 items that evaluate eight conceptual domains: general health (GH), physical functioning (PF), mental health (MH), role limitations - physical (RP), role limitations - emotional (RE), vitality (VT), bodily pain (BP), and social functioning (SF) [38]. Two summary measures known as the Physical Health Component Score (PCS) and the Mental Health Component Score (MCS) are derived. All the scales and summary measures are scored on a 0–100 scale, with higher scores indicating better health. The SF-36 has been used extensively for chronic pain populations [39, 40]. Psychometric testing for Arabic and Vietnamese translations has been performed, albeit not for chronic pain diagnoses [41–45].

### The depression, anxiety and stress scale (DASS-21)

The DASS-21 is a short form version of the original 42-item DASS questionnaire, designed to evaluate the extent to which a participant experiences the core symptoms of depression, anxiety, and stress [46]. The DASS-21 contains seven questions, scored between 0 (‘did not apply to me at all’) and 3 (‘applied very much or most of the time’), pertaining to the 3 psychological measures (depression, anxiety and stress subscales). Responses to each of the seven items are added to yield a total subscale score (/21). To facilitate comparison with the full DASS, each subscale score is doubled (/42) and interpreted as such [46]. The DASS-21 has been validated in clinical chronic pain samples [47] and undergone psychometric testing for Arabic and Vietnamese translations in other settings/conditions [48, 49].

### Statistical analysis

An a priori power analysis indicated a sample size of 80 participants would have 80% power to detect a correlation of *r* = 0.31 (representing a moderate correlation or higher), for each ethnocultural sub-group, while a total sample of 240 participants would provide sufficient power to detect a correlation as low as *r* = 0.18. Data were analysed using the Statistical Package for the Social Sciences, Version 24 [50]. Descriptive statistics for the entire sample and individual communities were computed as means (SD) for continuous data and % for categorical data.

#### Reliability

Test-retest reliability was assessed with Intra-class Correlation Coefficient (ICC 3,1) (two-way mixed-effects model, single measures, absolute agreement) and values are presented for each community and the entire sample, with 95% confidence intervals (Table 2). ICC’s were interpreted according to excellent (> 0.90), good (0.75 to 0.9), moderate (0.5 to 0.75), and poor (< 0.5) [51]. To quantify the precision of scores for each disk, the standard error of measurement (SEM) was calculated as SD√1-ICC and presented with the ICC for the entire sample [51, 52]. The minimum detectable change at 95% confidence (MDC_95_) was calculated as 1.96 *SEM*√2 to determine the magnitude of change that would exceed the threshold of error for each disk at the 95% confidence level [51, 52].

#### Construct validity

Construct validity was determined by correlating the placement of the PRISM ‘pain’ disk/SPS against validated Arabic and Vietnamese versions of various psychometric instruments. As the BPI measures pain severity and interference, it was anticipated that higher BPI scores would be associated with lower PRISM SPS. Further, higher general health and well-being that included physical, emotional, and social functions measured on the SF-36 were anticipated to be associated with higher PRISM SPS scores. Finally, the presence of higher negative emotional symptoms (depression, anxiety and stress) measured using the DASS-21 were expected to correlate with lower SPS. Correlations were analysed using either Pearson’s correlation, or Spearman’s rank correlation in the case of non-normally distributed data [53]. Correlations were interpreted according to strong (> 0.5), moderate (0.3 to 0.5), and weak (< 0.3) [54].

#### Cut off points for ‘high’, ‘medium’ and ‘low’ suffering

As for previous studies, we investigated the discriminative ability of previously suggested categories of suffering (high, medium and low) [16, 21]. ‘High’ suffering reflected a degree of overlap between pain and the self (SPS < 6.0 cm), ‘medium’ suffering (SPS 6.0 to 13 cm), and low suffering (SPS > 13 cm). Using a one-way ANOVA, mean scores for SPS, BPI, SF-36 and DASS subscale scores were compared across each suffering category. A Bonferroni post-hoc test was applied to control for multiple testing.

#### Qualitative analysis

Content validity was assessed by analysing participant responses for the placement of the ‘pain’ disk and the five additional disks, using thematic analysis [55]. Verbatim responses were translated by a NAATI accredited interpreter from the source language (Arabic, Assyrian or Vietnamese) to English for analysis. First, participant responses for each disk were coded for each ethnocultural community. Codes were sorted repeatedly as commonalities of meaning from successive participant responses emerged. Codes were then grouped into broader categories until main-themes were identified by an experienced qualitative researcher with clinical expertise in chronic pain [55]. Self-pain-separation distances were triangulated with the emergent themes to explore meanings associated with positioning of the pain disk.

## Results

### Demographics

Overall 251/302 (83%) of potential participants consented to participate and completed baseline assessments (Fig. 1). All Assyrian participants were bilingual, and 93% requested Arabic translations of standardised questionnaires and the remaining 7% requested English versions. Table 1 displays the sociodemographic characteristics of the sample and each ethnocultural community. All participants presented with neuromusculoskeletal pain, with an average duration of 9.6 years. Symptoms were constant for 81% of the sample and intermittent for the remaining 19%. Eighty percent of participants reported pain as affecting three or more areas in their body according to the pain diagram [56], with the most common locations over the back, neck and knees. The Vietnamese community had a longer duration in Australia and identified less as refugees compared with the Arabic and Assyrian communities. Psychometric profiles differed across the communities (Table 1).

**Table 1:** Participant Demographic Characteristics

	Arabic (*n* = 83)	Assyrian (*n* = 85)	Vietnamese (*n* = 83)	Sample (*n* = 251)
Age (years)	51.8 (10.2)	56.6 (9.7)	57.1 (11.7)	55.2 (10.8)
Gender, (*n*) Male:Female	22:61	29:56	24:59	75:176
Years in Australia	11.8 (11.5)	12.1 (10.4)	23.1 (9.3)	15.5 (11.6)
Migration circumstances *n* (%)				
Voluntary migrant	25 (30)	21 (25)	47 (57)	93 (37)
Refugee	58 (70)	64 (75)	36 (43)	158 (63)
Marital status - Married *n* (%)	61 (74)	60 (71)	45 (54)	167 (67)
Level of education, n (%)				
No school	3 (4)	11 (13)	3 (3.5)	17 (7)
Primary	15 (18)	19 (22)	22 (27)	56 (22)
Secondary	37 (44)	32 (38)	45 (54)	114 (45)
Tertiary	28 (34)	23 (27)	13 (15.5)	64 (26)
Duration of Pain (years)	10.5 (8.0)	9.9 (8.3)	8.3 (6.3)	9.6 (7.6)
Work status, *n* (%)				
Full or part-time work	1 (1)	2 (2)	9 (11)	12 (5)
Unemployed due to pain	63 (76)	56 (66)	43 (52)	162 (65)
Retired	3 (4)	9 (11)	16 (19)	28 (11)
Carer or domestic role	7 (8%)	7 (8%)	10 (12)	26 (10)
Other	9 (11%)	11 (13%)	5 (6%)	23 (9)
Receiving pension/benefit, *n* (%)	81 (98%)	81 (95%)	72 (87%)	230 (92)
Pension or benefit type				
Disability, *n*	19	25	18	62 (25)
Unemployment, *n*	48	39	27	114 (45)
Age pension, *n*	4	12	14	30 (12)
Carer pension / other, *n*	10	5	13	24 (10)
BPI (Pain Severity) /10	7.3 (1.9)	6.9 (1.9)	6.2 (1.9)	6.8 (1.9)
BPI (Pain Interference) /10	7.5 (1.9)	7.1 (1.9)	6.3 (2.3)	6.9 (2.1)
PRISM SPS /27	4.0 (4.6)	5.0 (4.9)	7.2 (6.8)	5.4 (5.6)
DASS-Depression /42	28.2 (10.5)	25.1 (11.1)	23.3 (13.1)	25.6 (11.8)
DASS-Anxiety /42	25.8 (10.7)	19.7 (11.6)	19.9 (11.5)	21.8 (11.6)
DASS-Stress /42	30.5 (8.9)	25.9 (9.9)	22.7 (10.6)	26.4 (10.3)
SF-36 Physical Component Summary^a^	31.7 (6.6)	32.7 (7.0)	33.7 (6.9)	32.7 (6.9)
SF-36 Mental Component Summary^a^	29.0 (9.7)	34.7 (10.6)	33.8 (12.2)	32.5 (11.1)
SF-36 Physical Functioning^a^	27.2 (25.6)	28.9 (23.5)	33.7 (22.6)	29.9 (24.0)
SF-36 Role Physical^a^	25.8 (21.8)	32.2 (24.0)	32.5 (24.1)	30.2 (23.5)
SF-36 Bodily Pain^a^	16.6 (14.9)	22.6 (16.2)	28.6 (19.1)	22.6 (17.5)
SF-36 General Health^a^	21.4 (17.8)	31.0 (19.5)	25.2 (19.3)	25.9 (19.2)
SF-36 Vitality^a^	21.7 (19.1)	27.9 (21.4)	29.9 (20.6)	26.5 (20.6)
SF-36 Social Functioning^a^	34.5 (25.4)	45.4 (24.0)	44.6 (25.7)	41.5 (25.4)
SF-36 Recreation^a^	28.6 (26.3)	40.6 (27.5)	38.4 (27.9)	35.9 (27.6)
SF-36 Mental Health^a^	29.3 (19.4)	32.8 (20.7)	40.1 (23.2)	35.9 (21.6)

*Values are presented as Mean (Standard Deviation) unless otherwise indicated. n* Number of participants, *%* Percentage within the group, *BPI* Brief Pain Inventory, *SPS* Self-Pain-Separation, *DASS* Depression, Anxiety and Stress Scale, *SF-36* Short Form 36

^a^All scores for SF-36 are calculated out of 100

### Reliability

From 251 participants, 238 participants completed the PRISM+ assessment on two occasions. The mean baseline SPS on occasion 1 was 5.5 cm (±5.7), while repeat SPS was 5.2 cm (±5.2). Paired samples t-tests revealed there was no difference between SPS means between occasions [t (237) = 0.23, *p* = 0.319). The ICC (3,1) for individual PRISM disks are displayed in Table 2. The ICC (3,1) for SPS was 0.78 (95% CI 0.73 to 0.83) with an MDC_95_ of 3.31 cm. Thus, a change in SPS of > 3.31 cm is required to be 95% certain that a real change in pain-related suffering has occurred between measurement occasions, rather than from test-retest variability or measurement error. Similarly, the ‘resource’ disks demonstrated moderate to good reliability, with MDC_95_ scores ranging from 6.35 to 2.29 cm (Table 2).

**Table 2:** Test-Retest reliability ICC (3,1, absolute agreement)

PRISM Disk	CALD Community Sub-groups			Overall ICC (3,1)^*˄*^ r (95% CI), *n*	Overall SEM	Overall MDC_95_
	Arabic r (95% CI)^˄^, *n*	Assyrian r (95% CI) ^˄^, *n*	Vietnamese r (95% CI) ^˄^, *n*			
PRISM Pain	0.76 (0.65 to 0.84), 80	0.65 (0.50 to 0.76), 78	0.82 (0.73 to 0.88), 80	0.78 (0.73 to 0.83), 238	1.19	3.31
PRISM Spirituality	0.74 (0.62 to 0.82), 79	0.63 (0.48 to 0.75), 78	0.71 (0.57 to 0.80), 78	0.71 (0.64 to 0.77), 235	1.89	5.25
PRISM Family	0.66 (0.52 to 0.77), 79	0.77 (0.66 to 0.85), 78	0.54 (0.36 to 0.67), 80	0.67 (0.60 to 0.74), 237	1.58	4.39
PRISM Spouse	0.75 (0.62 to 0.84), 62	0.86 (0.78 to 0.91), 67	0.91 (0.86 to 0.95), 59	0.88 (0.84 to 0.91), 188	0.83	2.29
PRISM Recreation	0.75 (0.63 to 0.83), 80	0.71 (0.59 to 0.81), 78	0.63 (0.48 to 0.74), 80	0.70 (0.63 to 0.76), 238	2.26	6.25
PRISM Work	0.68 (0.54 to 0.78), 80	0.79 (0.68 to 0.86), 78	0.65 (0.50 to 0.76), 80	0.71 (0.64 to 0.77), 238	2.29	6.35

*CALD* Culturally and Linguistically Diverse; ˄all ICC *p* < 0.0005; *n* number of participants includes only participants for which this disk applied

### Construct validity

All the a priori hypotheses were supported, with significant correlations between SPS for the PRISM and assessment instruments respectively, in the directions hypothesised (Table 3). Moderate correlations were observed between PRISM SPS and: BPI pain severity and interference subscales respectively; the SF-36 PCS and MCS subscales respectively; and the DASS total and subscale scores respectively. Most individual items from the SF-36 demonstrated moderate correlations with the PRISM SPS, with the strongest correlation observed for SF-36 MH scores (*r* = 0.402, *p* < 0.001). Weak correlations were observed with the PRISM SPS for individual items of the BPI and the SF-36 recreation item. Table 3 summarises the correlations, for the whole sample and each community.

**Table 3:** Correlation of PRISMSelf-Pain -Separation with Clinical Outcome Measures

	Overall Sample (*n* = 251)	CALD community subgroups		
		Arabic Community (*n* = 83) (*ρ*)	Assyrian Community (*n* = 85) (*ρ*)	Vietnamese Community (*ρ*)
BPI Pain Severity	*r* = −0.34**	− 0.31*	−0.35**	*r* = − 0.25*
BPI Pain Interference	*r* = − 0.34**	−0.29*	− 0.33*	− 0.31*
BPI Pain ‘Worst’	ρ = − 0.28**	− 0.27*	− 0.14	−0.36**
BPI Pain ‘Least’	ρ = − 0.25**	− 0.20	− 0.39**	− 0.09
BPI Pain ‘Average	ρ − 0.30**	− 0.30*	− 0.31*	−0.16
BPI Pain ‘Right Now’	ρ = − 0.27**	−0.28*	− 0.29*	−0.21
SF-36 Physical Component Summary	*r* = 0.31**	0.35*	0.33*	0.26*
SF-36 Mental Component Summary	*r* = 0.36**	0.26*	0.47**	0.31*
SF-36 Physical Functioning	ρ = 0.34**	0.25*	0.45**	0.29*
SF-36 Role Physical	ρ = 0.36**	0.33**	0.35**	0.38**
SF-36 Bodily Pain	ρ = 0.36**	0.39**	0.38**	0.26*
SF-36 General Health	ρ = 0.30**	0.37**	0.38**	0.26*
SF-36 Vitality	ρ = 0.34**	0.23*	0.40**	0.30*
SF-36 Social Functioning	ρ = 0.30**	0.28*	0.38**	0.24*
SF-36 Recreation	ρ = 0.27**	0.16	0.34**	0.26*
SF-36 Mental Health	*r* = 0.40**	0.33*	0.50**	0.33*
DASS-Depression	ρ = −0.37**	−0.48**	−0.35**	−0.30*
DASS-Anxiety	ρ = − 0.31**	−0.32*	− 0.32*	−0.20
DASS-Stress	ρ = − 0.39**	−0.44**	− 0.38**	−0.27*

*BPI* Brief Pain Inventory, *SF-36* Short Form 36, *DASS* Depression, Anxiety and Stress Scale; *r* denotes Pearson’s test performed, *ρ* denotes Spearman’s test performed, *p < 0.05 level; **p ≤ 0.001

### Cut off points for ‘high’, ‘medium’ and ‘low’ suffering

Table 4 displays the discriminative ability of PRISM for categorising suffering as high, medium, or low. Mean scores for BPI, SF-36 and DASS subscale scores are presented for each suffering category. Between group comparisons demonstrated the SPS category of low suffering for PRISM differed significantly from moderate suffering, and high suffering respectively, for all assessment tools (*p* < 0.001). The SPS categories of medium and high suffering from PRISM did not significantly differ from each other for any assessment tool (*p* > 0.05).

**Table 4:** Between group comparisons for SPS for the three PRISM groups using ANOVA

	High SPS < 6 cm *n* = 160	Medium SPS 6 cm – 13 cm *n* = 63	Low SPS > 13 cm *n* = 28	Difference of the PRISM groups: Mean (95% CI)			F (2, 248)
				Low vs Medium	Low vs High	Medium vs High	
Pain Severity	7.32 ± 1.82	6.30 ± 1.64	5.43 ± 1.77	−0.87 (−1.84 to - 0.10) NS	−1.90 (−2.77 to −1.02)**	−1.03 (−1.66 to − 0.39)**	17.83**
Pain Interference	7.53 ± 2.00	6.40 ± 1.87	5.02 ± 1.89	− 1.38 (− 2.45 to − 0.31) NS	−2.50 (−3.47 to − 1.54)**	− 1.13 (− 1.83 to − 0.42)**	22.90**
SF-36 PF	25.18 ± 22.40	36.68 ± 23.19	45.89 ± 18.47	9.20 (−2.96 to 21.36) NS	20.71 (9.74 to 31.67)**	11.51 (3.54 to 19.47)*	13.75**
SF-36 RP	23.67 ± 18.95	40.55 ± 22.91	49.48 ± 21.15	8.94 (−2.15 to 20.02) NS	25.81 (15.81 to 35.81)**	16.88 (9.62 to 24.14)**	29.12**
SF-36 BP	18.74 ± 15.13	27.81 ± 15.19	37.47 ± 15.08	9.66 (1.37 to 17.95)*	18.74 (11.26 to 26.21)**	9.07 (3.64 to 14.50)**	22.30**
SF-36 GH	22.49 ± 17.08	29.45 ± 19.42	41.01 ± 16.84	11.56 (1.89 to 21.23)*	18.52 (9.80 to 27.24)**	6.96 (0.63 to 13.30)*	14.43**
SF-36 SF	36.28 ± 22.64	47.90 ± 25.04	61.27 ± 18.76	13.37 (0.84 to 25.90)*	24.99 (13.69 to 36.29)**	11.62 (3.41 to 19.82)*	17.01**
SF-36 RE	30.12 ± 25.27	43.50 ± 28.76	57.76 ± 25.65	14.26 (−0.1 to 28.62) NS	27.65 (14.70 to 40.60)**	13.39 (3.99 to 22.79)*	16.19**
SF-36 MH	29.54 ± 19.39	44.25 ± 18.56	56.7 ± 17.57	12.21 (1.82 to 22.61)*	26.93 (17.55 to 36.31)**	14.72 (7.91 to 21.53)**	31.45**
SF-36 V	22.09 ± 18.02	32.01 ± 18.88	44.44 ± 16.62	12.42 (2.52 to 22.33)*	22.35 (13.41 to 31.28)**	9.93 (3.44 to 16.41)*	21.32**
DASS-Depression	28.38 ± 11.18	22.24 ± 10.80	17.30 ± 7.77	−4.94 (−10.83 to 0.95) NS	−11.08 (− 16.39 to −5.76)**	−6.14 (− 10 to −2.28)**	16.72**
DASS-Anxiety	24.15 ± 11.37	19.46 ± 10.99	14.53 ± 8.03	− 4.93 (− 10.23 to 1.07) NS	−9.62 (− 15.03 to − 4.21)**	−4.67 (−8.61 to − 0.76)*	11.26**
DASS-Stress	29.08 ± 10.13	23.08 ± 8.37	18.80 ± 6.69	−4.29 (− 9.43 to 0.85) NS	−10.29 (− 14.92 to − 5.65)**	−6.0 (− 9.36 to − 2.63)**	19.63**

Data are presented as Mean ± SD unless otherwise stated; ** *p* ≤ 0.001; * *p* < 0.05

*NS* Not significant (*p* >0.05, *SPS* Self-Pain-Separation, S*F-36* Short Form 36, *PF* Physical Functioning, *RP* Role Physical, *BP* Bodily Pain, *GH* General Health, *SF* Social Functioning, *RE* Recreation, *MH* Mental Health, *V* Vitality, *DASS* Depression, Anxiety and Stress Scale

### Content validity

Representative participant responses for the ‘pain’ and additional disks are displayed in Table 5. Qualitative analysis of participant responses for the PRISM pain disk identified three main themes: (1) ‘threat appraisal’, (2) ‘expectations/controllability’, and (3) ‘intrusiveness’. Triangulating the themes with the SPS categories revealed a continuum of responses and sub-themes that were unique for each SPS category. For participants with high pain-related suffering (low SPS), threat appraisal was underpinned by the threat to personal identity, a sense of captivity, and fear for the future. Conversely, participants with low pain-related suffering (high SPS) appraised the threat of pain with acceptance and resilience. For those participants with a medium score, a ‘transitioning’ subtheme emerged between recognition of a need for acceptance, and a struggle to do so. In a similar manner, the theme of ‘expectations/controllability’ reflected a transition from powerlessness and uncertainty (high suffering) to acceptance and familiarity of symptoms (low suffering). The theme of pain intrusiveness emerged only for participants in the high and medium suffering groups, as participants reflected on the interference with aspects of daily living and personal efforts to ‘be careful’ not to ‘make my situation worse’ (Table 5).

**Table 5:** Participant responses for placement of the Pain disk

	High Suffering Self-Pain-Separation < 6 cm	Medium Suffering Self-Pain-Separation 6 cm – 13 cm	Low Self-Pain-Separation > 13 cm
Threat appraisal	*Identity* *[SPS 0.2 cm] “The pain affects every part of my being. I am suffering with the thing … the thing it is like it is eating me all. It consumes all of me. There is no escape” (Assyrian)* *[SPS 3.6] “The pain has changed me. I am not the man [I was]. I am weak and torn apart. I can’t even look in the mirror sometimes to see what it has made of me” (Arabic)* *Captive* *[SPS 3.2 cm] “It is like you are trapped. The pain is on all sides and I can’t escape from it. Nothing I can do can free me from this pain. It is my prison” (Arabic)* *Fear* [SPS 5.3] “*It is too much. I try not to tell other people about my pain, because I feel bad every time I talk about it. Almost about to cry, the pain makes me afraid for my life”* (Vietnamese)	*Transitioning* *[SPS 11.7] "The truth is I must accept and learn to live with this pain. I mean this part of me hurts, this is not foreign to me. Also, say both my legs hurt. I do have this pain but I have to move [pause]. I have to try and move it because in the end, these are mine and mine for good (Assyrian)* *[SPS 7.9] “The pain is unnatural. It is not just physical, but emotional. It feels heavy on my whole person. It diminishes the happiness. I am tired with the thing” (Arabic)* *[SPS 8.1] “I have pain in my back and in my legs. The doctors say I will not escape this pain and I must learn to live with it. I am trying but 50/50” (Vietnamese)*	*Acceptance and Resilience* *[SPS 13 cm] “The pain is there … I know it is there, but I am trying not to let it change me, or my life. I do what I can” (Assyrian)* *[SPS 13.6] “The pain is in the middle. I think other things come first. It is in the centre” (Arabic)* *[SPS 23 cm] “You have to help yourself. This does not mean I do not feel pain, but I do everything to give me power to control the pain and I make choices how I will live” (Vietnamese)*
Expectations/ Controllability	*[SPS 1.0] “The pain is constant. It takes your breath away, I have no idea why I suffer with it, I have trouble with how to move many times” (Assyrian)* *[SPS 0.9] “The pain makes me powerless and unable to do anything. I ‘ve got joint pain and the pain is unbearable” (Arabic)* *[SPS 1.2 cm] “Pain comes before everything. It only gets worse and comes to take more and more of me. I don’t know how to stop it. One day it may take all of me” (Vietnamese)*	*[SPS 10.5] “It is there but I try not to think on it too much. I think I need to help myself in this way [pause]. To not let it control or take over” (Assyrian)* *[SPS 8.8] “It feels like the pain is closing in on me. I try to push it away. I use too many tablets to help the pain. This is not always enough. I know I need to learn to control it” (Arabic)* *[SPS 7.2] “The pain is there. I don’t know why, but I notice if I use hot oil or exercises it gets better, so I can stop it sometimes from taking me too much” (Vietnamese)*	*[SPS 14.5] “I do have pain [pause]. But I have lived with this for a long time now. What can you expect when you get old and the bones are weak and the pain can come. But I expect this. I know this, like a friend [laugh]”. (Vietnamese)* *[SPS 22.8] “I know I will never be without pain. How can I expect this? As the body is older it is natural, so I think I do not worry for this and try to live my life my way” (Vietnamese)*
Intrusiveness	*[SPS 3.8 cm] “The pain is part of me. It touches each part of my life, my family, my work, my prayer [pause] even I can’t do this fully with the pain I have. I suffer with this in all my life” (Arabic)* *[SPS 1.4 cm] “I think because I have the pain very much [pause]. Pain interferes with my life and creates difficulty in my life” (Vietnamese)*	*[SPS 10.3] “The pain does affect me [pause]. The heavy jobs have affected my muscles and my nerves and as I am getting older my mind too [pause]. Even now I feel it stronger each day, but I try to look after myself, be careful and not make my situation worse” (Assyrian)* *[SPS 6.5 cm] “My injury has affected my life. It does not let me live like before. I have to be careful with everything to control the pain I feel” (Vietnamese)*	*[SPS 14.3] “The pain is above me. It goes up and down. Sometimes it can be overwhelming, but I have to push it away and not let it affect my mind too much” (Assyrian)*

*SPS* Self-Pain-Separation

Qualitative analysis of the five additional disks yielded four key themes (‘relative to the collective’, ‘meaning and sense of worth’, ‘role expectations’, and ‘physicality’) focused around a broader concept of the ‘relational self’ or self as embodied by socio-cultural-spiritual values and interconnections with significant others (Table 6).

**Table 6:** Themes arising from placement of the Additional discs

	Work	Recreation	Spouse	Family	Spirituality
Relative to the collective	*“Work, helping others, looking after the family, the house, this is important. I volunteer, I do this because I want to do something to keep my mind and body active” (Assyrian SWS 8.1)*	*“I know that being with others helps me [pause] it keeps me going [pause] and I learn and share with others” (Vietnamese, SRS 3.5)* *“I feel the support of my community. We share, we help each other. My troubles seem less when I am with people” (Assyrian, SRS 4.9)* *“I don’t like to go out. I don’t want to see people [pause]. I think things have affected me so, I don’t feel comfortable with this in my life” (Arabic, SRS 27.0)*	*“My husband, he is next to me. We support each other [pause]. If there is caring between husband and wife, that is what matters” (Assyrian, SSpS 4.5 cm)*	*“This is hard because my children and I are not close [pause]. They don’t listen to me anymore [pause]. I think maybe because of the pain. I am now weak/ [seen as weak by them]”. (Vietnamese, SFS 15.1)* *“You know the pain it changes you. It changes how you feel with others and how I can talk and do things with my family. It is not the same. They do not want to be around that” (Arabic, SFS 20.4)*	*“I am Assyrian [pause]. We Assyrians, you know, spirituality, religion, God [pause], it is like the breath we breathe [pause], it is within us” (Assyrian, SSS 2.4)* *“This is me [pause], it is part of me [pause]. I am Mandaean and for us, this is more than just pray, it is who we are” (Arabic, SSS 0.1)*
Meaning & sense of worth	*“Should be my life [pause] to keep the house and cook the meal and be proud of this, but no matter how I try the pain it pierces me, and I collapse [pause] I am trying” (Vietnamese, SWS 18.5)* *“It is part of my life, who I am and was raised to be. Even when I cannot, the will is strong” (Assyrian, SWS 2.5)*	*“In our community we are close [pause], we help each other, try to do this [pause], to be with people. Think it helps” (Assyrian, SRS 10.7)*		*“They are your heart [pause]. My children make me strong [pause], are special to me” (Vietnamese, SFS 0.7)* *“My family are my purpose, my life.” (Assyrian, SFS 6.4)* *“My children are my life. My breath and the reason I am here today. They are why I want to change my life”* *(Arabic, SFS 3.1)*	*“I think this helps keep me centred. It guides how you can view life and what happens” (Arabic, SSS 5.9)* *“I am Buddhist [pause], this is so calm for me. It helps me to focus about what is important [pause] find meaning in life and this I find for me” (Vietnamese, SSS 1.3)* *“I try to believe. I want to give myself to this, but I am lost. I find this too hard on me now” (Arabic, SSS 13)* *“I don’t believe [pause]. The things that have happened to me [pause]. I can’t anymore” (Arabic, SSS 25.2)*
Role expectations	*“work is important to me [pause] I should be able to provide for my family [pause]. This is who I am [pause], but the pain [pause], it limits me” (Assyrian, SWS 5.4)* *“My work is to my family and my husband [pause]. I care for them [pause]. This is who I am [pause]. I like things to be a certain way, and I try to maintain that, it is who I am” (Assyrian, SWS 6.3)* *“I would love to put it on top of me [pause]. Because it is important you know [pause], it should be. I am a proud man. But this is too hard [pause]. So, it is a long way [pause]. This is the truth of the situation” (Arabic, SWS 27.0)*		*“My husband he is a good man. He has been by my side through it all [pause], even when I thought I was going to die [pause], he is my strength.” (Arabic, SSpS 4.7)* *“Our relationship is difficult. I am not who I should be for her. I think sometimes she would be better off without me. but we are not close like before [pause], how can we be, when I am like this” (Arabic, SSpS 12.2)*	*“My family look after me. Even the grandchildren see I am in pain and walk with me”* *(Vietnamese SFS 4.6 cm)"* *“This is my daughter. My daughter is very important to me. She lives with me and helps me a lot [pause], she is a good girl” (Assyrian, SFS 8.0)*	*“my belief is that God is the answer to everything. I turn to him when I am stressed or upset or in pain and believe that with his help, I will be cured” (Assyrian, 8.3 cm)*
Physicality	*“It is impossible to work as I want. I cannot lift a finger without the pain piercing right through me” (Assyrian SWS 22.9)* *"I used to carry heavy machinery, and this is how my pain started. Even my hands are hurting, I cannot carry 1 k of anything with my hands, even when I pour myself tea from the teapot, even that is painful (SWS 22.4)* *“I try to do what I can do. But I am not strong like before. It stops me a lot” (Assyrian, SWS 22.9)*	*“I can’t do things I used to. Believe me I was a sport person before this. I could run and play soccer and be social, but now I can hardly even walk” (Arabic, SRS 13.8 cm)* *“My pain stops me from sitting or walking or being with people for long periods. I find this hard” (Assyrian, SRS 18.2 cm)*		*“I have a new baby you know. He is 4 months old. I can hardly hold him, but he is my blood. my family are my heart” (Arabic, SFS 4.0 cm)*	*“This is important … however the pain interferes with my ability to attend church. I am Catholic, and I cannot pray properly, because of my knees. This makes it hard”* *(Vietnamese, SSS 11.8 cm)*

*SWS* Self-Work-Separation, *SRS* Self-Recreation-Separation, *SSS* Self-Spirituality-Separation, *SSpS* Self-Spouse-Separation, *SFS* Self-Family-Separation

Participants from each community recognised work as valued as part of a collectivist culture (relative to the collective), part of their identity (sense of worth), and/or relating to fulfilment of a sociocultural role (role expectations). Similarly, the recreation disk portrayed narratives communicating sociocultural values of community engagement and a sense of worth attributed to being part of a collective.

Spirituality was an important disk for all three communities despite different inter- and intra-cultural religious affiliations. Participants reflected on the connectedness it brought them with others from their community (relative to the collective), the perspective and meaning it offered to their pain experience (meaning and sense of worth) and their relationship/ role relative to their deity (role expectations). Finally, the spouse and family disks were interconnected and against personal and community role expectations. Participant accounts portrayed tensions between role expectations to act as the ‘giver’ (e.g. caregiver) and reliance/dependence on care or others. Further, family and spouse disks encouraged reflection on values of emotional connectedness and sense of meaning/purpose in life. For all disks, disk appraisal and placement were interconnected with their physicality, perceived ability to engage with each disk, and therefore highly influenced by pain.

## Discussion

Comprehensive pain assessment requires holistic exploration of biological, psychological and sociocultural dimensions of the pain experience [57]. However, limited psychometric testing and/or availability of commonly used assessment tools mean the pain experience may be inadequately represented for CALD communities [2, 3, 58, 59]. This includes for Assyrian, Arabic and Vietnamese communities for whom psychometric testing of translated tools (BPI, DASS and SF-36) has not been undertaken in a chronic pain cohort. Our results support the reliability and validity (construct and content) of the PRISM+ among Arabic-speaking, Assyrian, and Vietnamese patients with chronic pain, which provides a novel alternative to traditional pain questionnaires. Importantly, the pictorial and holistic nature of the PRISM+, circumvents many of the challenges of cross-cultural pain assessment, while eliminating potential problems associated with numerical and scale assessments among low literacy cohorts or where different cultural interpretations (such as the representational value of numbers) might influence their validity [2, 60].

Our study revealed CALD communities consistently position the ‘pain’ disk relative to the ‘self’ (SPS) between occasions with good overall reliability (ICC 0.78) and within each ethnocultural group (Table 2). Such findings are consistent with correlations observed in other applications of the PRISM. For example, previous studies that included participants with diabetes, orofacial pain, and chronic pain diagnoses demonstrated reliability coefficients/correlations between 0.79 and 0.99 [12, 15, 21]. Building on previous research [17, 21, 22], our results also provide evidence that the PRISM is a useful tool for detecting change over time. The MDC_95_ estimates for SPS of 3.31 cm provides healthcare providers with 95% certainty that a change in SPS of 3.4 cm or more can be attributed to real change in pain-related suffering [51]. To our knowledge this is the first report of MDC_95_ with potential to enhance interpretation of the ‘illness’ scores from PRISM+ in future applications as a treatment outcome [17, 21, 22, 61].

Our investigation of the construct validity of PRISM demonstrated ‘pain’ disk placement (SPS) correlated significantly in the anticipated directions with other tools designed to explore the multiple dimensions of pain that included for pain severity and interference (BPI), quality of life (SF-36), and negative emotional state (DASS) (Table 3). However, the strength of the correlations was only moderate (0.31 to 0.40). This is consistent with correlations observed in previous applications [12, 24], and reflects the novelty of PRISM+. Specifically, it can be argued such data support the conclusion that PRISM+ measures a different construct to symptom, disability and quality of life tools.

It has been consistently argued that PRISM is a measure of illness-related suffering [11, 21, 22], and our qualitative analysis, provides evidence consistent with this supposition. Suffering is considered to arise from a threat to one’s identity, collectively encompassing physical symptoms, psychological, and existential/spiritual distress [8, 20, 23]. Thus, pain-related suffering reflects more of the meaning of chronic pain to the individual, rather than intensity or function alone, as demonstrated in the strength of the correlations we observed (Table 3). The themes of ‘threat appraisal’, ‘controllability’, and ‘intrusiveness’, that emerged from our thematic analysis, all relate to the construct of suffering [8, 20, 23, 62]. Further, if suffering reflects an imbalance between perceived threat and the resources to cope with arising threats [62], then narratives of distress and coping would be expected to vary across high, medium and low suffering states. Indeed, ‘fear’, ‘captive’, and ‘threat to identity’ were characteristic of distress in the ‘high suffering’ category, while accounts of ‘acceptance and resilience’, were portrayed by those from the ‘low suffering’ category. Thus, our quantitative and qualitative data adds to the growing body of evidence validating PRISM as a tool for exploring suffering [8, 12, 21, 22, 63].

In addition to validation of the ‘pain’ disk, the current study demonstrated moderate to good reliability and content validity for the five additional disks. Despite necessarily limited instruction accompanying the task, participants were consistent in their interpretation of all additional disks (‘family’, ‘spouse’, ‘spiritualty’, ‘recreation’, and ‘work’), with ICC’s between 0.67 to 0.88, and all MDC_95_ estimates within a quarter of the total score (Table 2). The limited instruction accompanying the disks was critical for ensuring personal interpretations and narratives of each dimension. For example, the ‘spirituality’ disk prompted participants to reflect on where they would place spirituality in their lives, if relevant. Once positioned, the healthcare provider was able to explore meanings underpinning disk placement. Herein lies the clinical utility of the PRISM+ as a non-confrontational means for enquiring about cultural and religious values [6, 64].

PRISM+ as a tool for exploring sociocultural and religious values was substantiated by qualitative analysis. The narratives accompanying each additional disk placement were consistent with themes reported in previous qualitative research with Assyrian, Arabic-speaking and Vietnamese communities [6]. In the current study, participants interpreted each ‘additional disk in ways that reflected the value of traditional gender/cultural roles (e.g. *“I should be able to provide for my family”*), the importance of the family unit (eg. hierarchical relationships as in the case of the Vietnamese community: *“my children … don’t listen to me anymore … I am now weak”),* collectivist community approaches to life’s challenges (e.g. *“I feel the support of my community”)* and spiritual contributions to health and wellbeing*.* The consistency of these themes with in-depth qualitative research is important, as healthcare providers require focused responses in their time-limited clinical assessments, rather than lengthy phenomenological enquires [8]. Thus, the succinct social and cultural knowledge arising from the additional disks is likely to add value to the pain assessment, informing directions for treatment that incorporate the patient’s worldview and/or facilitates the adaptation of pain management approaches [7]. Potentially, the quantitative measure yielded by the self-additional disk-separation measure allows the patient and healthcare provider an opportunity for measuring changes in the positioning of important dimensions in one’s life, as may be intended with therapy [11]. For example, addressing the physicality dimension of spirituality through treatment may facilitate a reduction in the self-spirituality-separation. As such, the additional disks may provide patients and healthcare providers with a culturally specific treatment outcome measure.

A final advantage arising from the interview format of PRISM+ is the resulting therapeutic effects of patient engagement and rapport building, while also capturing salient personal information about sociocultural context [65, 66]. Unlike self-reported questionnaires, which are often perceived by patients to be impersonal and lengthy, the two-way interactive discussion of PRISM+ could provide a means for engaging patients if disks selected are tailored to the individual [8, 67, 68]. Indeed, orientation of medical assessments towards the sociocultural context of illness is known to enhance trust in patients from CALD communities, satisfaction, and adherence to treatment [69, 70]. While satisfaction with the PRISM+ was not formally evaluated in this study, the PRISM face to face interaction was well received by participants, easily interpreted, and it has successfully been used to guide cultural adaptation of treatment [7]. As such, the PRISM is a valuable alternative for engaging CALD patients in cross-cultural encounters, providing a foundation from which behavioural change can begin to occur.

### Study limitations

It is important to consider the findings of this study in the context of its limitations. First, only three CALD communities were included from patients who had been referred to a public health service for physiotherapy or pain management. As such, our cohort may not have captured participants with lower pain scores or participants with pain arising from non-musculoskeletal causes. Potentially this would influence the strength of certain associations and the generalisability of our findings to such cohorts. Second, since no gold standard of suffering has been established, construct validity could only be evaluated against existing validated questionnaires that measure constructs distinct from suffering. The choice of questionnaires was further constrained by the lack of reliable and valid translations of commonly used pain questionnaires. This likely accounts for some of the weaker correlations observed in this study. Third, since SEM was calculated from inferred stability of pain between occasions, conclusions about the responsiveness of PRISM+ to change over time in response to treatment remains to be compared to established tools. Further, the relationships between the themes identified in the qualitative analysis and formal assessments for pain coping and perceived threat was unable to be quantitatively established in the current design. Finally, the use of the additional disks as a culturally specific outcome measure from which directions for, and progress with treatment is yet to be substantiated. Thus, further research is needed to explore fully the utility of the PRISM+ within CALD communities and its relationship to other pain and biopsychosocial assessments to substantiate the broader use of the PRISM+ as an outcome measure.

## Conclusion

The PRISM+ demonstrated acceptable reliability and validity for assessing multiple dimensions of the chronic pain experience in Arabic-speaking, Assyrian and Vietnamese communities. The findings of this study add to the literature that promotes the use of a bio-psycho-sociocultural framework to understand the experience of pain in persons with chronic conditions. Finally, the PRISM+ provides healthcare providers with a novel method to explore pain narratives in culturally responsive ways that can be used as an alternative or to complement to other established pain instruments.

## Additional file

Additional file 1: The Pictorial Representation of Illness and Self Measure. A graphical representation of the Pictorial Representation of Illness and Self Measure (English version) (TIF 1960 kb)

## Abbreviations

BP: Bodily Pain
BPI: Brief Pain Inventory
CALD: Culturally and Linguistically Diverse
DASS: Depression Anxiety and Stress Scale
GH: General Health
HREC: Human Research Ethics Committee
ICC: Intraclass Correlation Coefficient
MCS: Mental Health Component Score
MDC_95_: Minimum detectable change at 95% Confidence level
MH: Mental Health
NAATI: National Australian Authority for Translators and Interpreters
PCS: Physical Health Component Score
PF: Physical Functioning
PRISM+: Pictorial Representation of Illness and Self Measure Plus
RE: Role Emotional
RP: Role Physical
SEM: Standard Error of Measurement
SF: Social Functioning
SF-36: Short-Form 36 Health Survey
SPS: Self-Pain Separation
SWSLHD: South West Sydney Local Health District
VT: Vitality
