# Supplementary figures and images for: Recognising ethnocultural diversity in chronic pain assessment: validation of the Pictorial Representation of Illness and Self Measure (PRISM) for use with culturally diverse communities
Source: Health Qual Life Outcomes. 2019 Apr 8;17:56. doi: 10.1186/s12955-019-1126-9 (PMC6454629; doi:10.1186/s12955-019-1126-9)

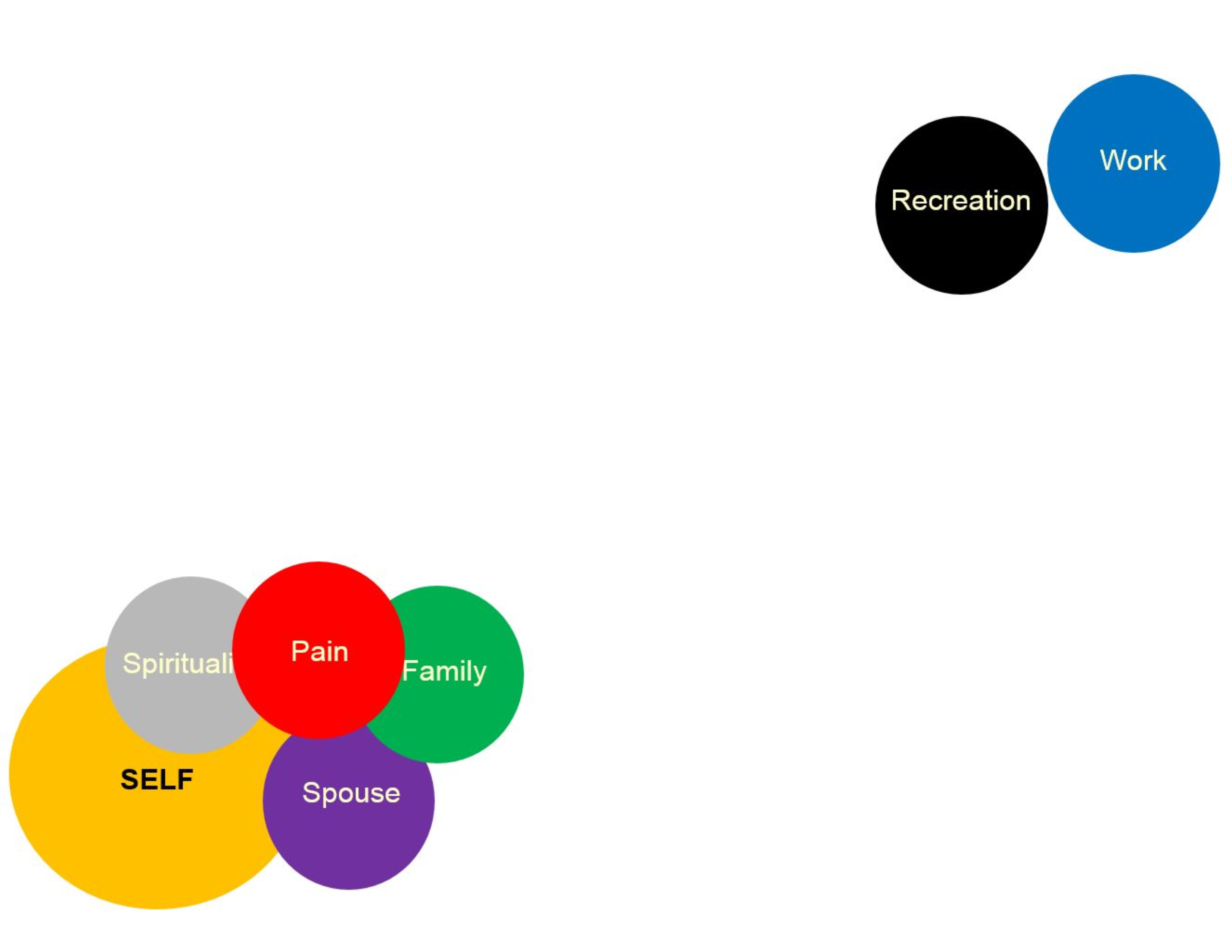

Supplement: Supplementary file 1 — The Pictorial Representation of Illness and Self Measure. A graphical representation of the Pictorial Representation of Illness and Self Measure (English version) (TIF 1960 kb) [file 12955_2019_1126_MOESM1_ESM.tif]
